# Electrochemical sensors based on metal nanoparticles with biocatalytic activity

**DOI:** 10.1007/s00604-022-05252-2

**Published:** 2022-04-02

**Authors:** Katarzyna Białas, Despina Moschou, Frank Marken, Pedro Estrela

**Affiliations:** 1 Centre for Biosensors, Bioelectronics and Biodevices (C3Bio), University of Bath, Bath, BA2 7AY UK; 2 Department of Electronic and Electrical Engineering, University of Bath, Bath, BA2 7AY UK; 3 Department of Chemistry, University of Bath, Bath, BA2 7AY UK

**Keywords:** Biosensor, Nanozyme, Point-of-care, Diagnostics, Theranostics, Nonenzymatic sensors

## Abstract

Biosensors have attracted a great deal of attention, as they allow for the translation of the standard laboratory-based methods into small, portable devices. The field of biosensors has been growing, introducing innovations into their design to improve their sensing characteristics and reduce sample volume and user intervention. Enzymes are commonly used for determination purposes providing a high selectivity and sensitivity; however, their poor shelf-life is a limiting factor. Researchers have been studying the possibility of substituting enzymes with other materials with an enzyme-like activity and improved long-term stability and suitability for point-of-care biosensors. Extra attention is paid to metal and metal oxide nanoparticles, which are essential components of numerous enzyme-less catalytic sensors. The bottleneck of utilising metal-containing nanoparticles in sensing devices is achieving high selectivity and sensitivity. This review demonstrates similarities and differences between numerous metal nanoparticle-based sensors described in the literature to pinpoint the crucial factors determining their catalytic performance. Unlike other reviews, sensors are categorised by the type of metal to study their catalytic activity dependency on the environmental conditions. The results are based on studies on nanoparticle properties to narrow the gap between fundamental and applied research. The analysis shows that the catalytic activity of nanozymes is strongly dependent on their intrinsic properties (e.g. composition, size, shape) and external conditions (e.g. pH, type of electrolyte, and its chemical composition). Understanding the mechanisms behind the metal catalytic activity and how it can be improved helps designing a nanozyme-based sensor with the performance matching those of an enzyme-based device.

**Graphical abstract:**

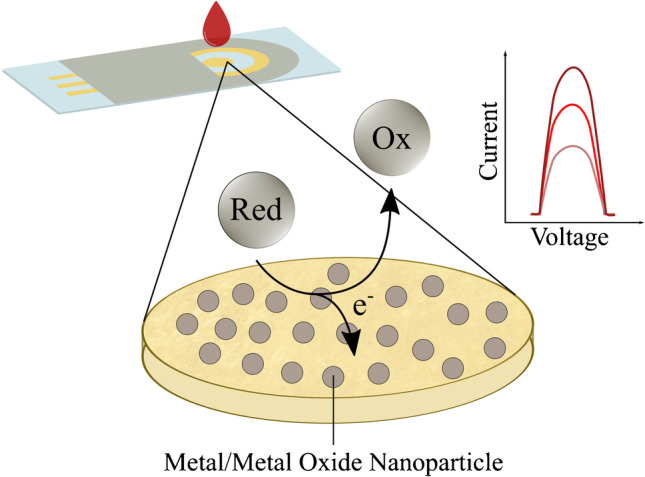

## Introduction

For many years, sensors have been present in nearly every aspect of life, including but not limited to healthcare, environmental protection, and food and safety inspection. Researchers worldwide have been working on translating standard laboratory-based assays into simple, affordable, and portable devices. Extensive research and rapid development of microfluidics and nanotechnology have opened the door to the design of lab-on-a-chip (LOC) devices — fully integrated miniaturised biochemical analysis laboratories with minimal sample usage and user intervention. Despite their small size, other characteristics such as sensitivity, selectivity, stability, and reproducibility do not need to be compromised. With broad knowledge and innovative technology, researchers can eventually design LOC-compatible biosensors with sensing characteristics matching or exceeding those of standard laboratory techniques.

**Fig. 1:**
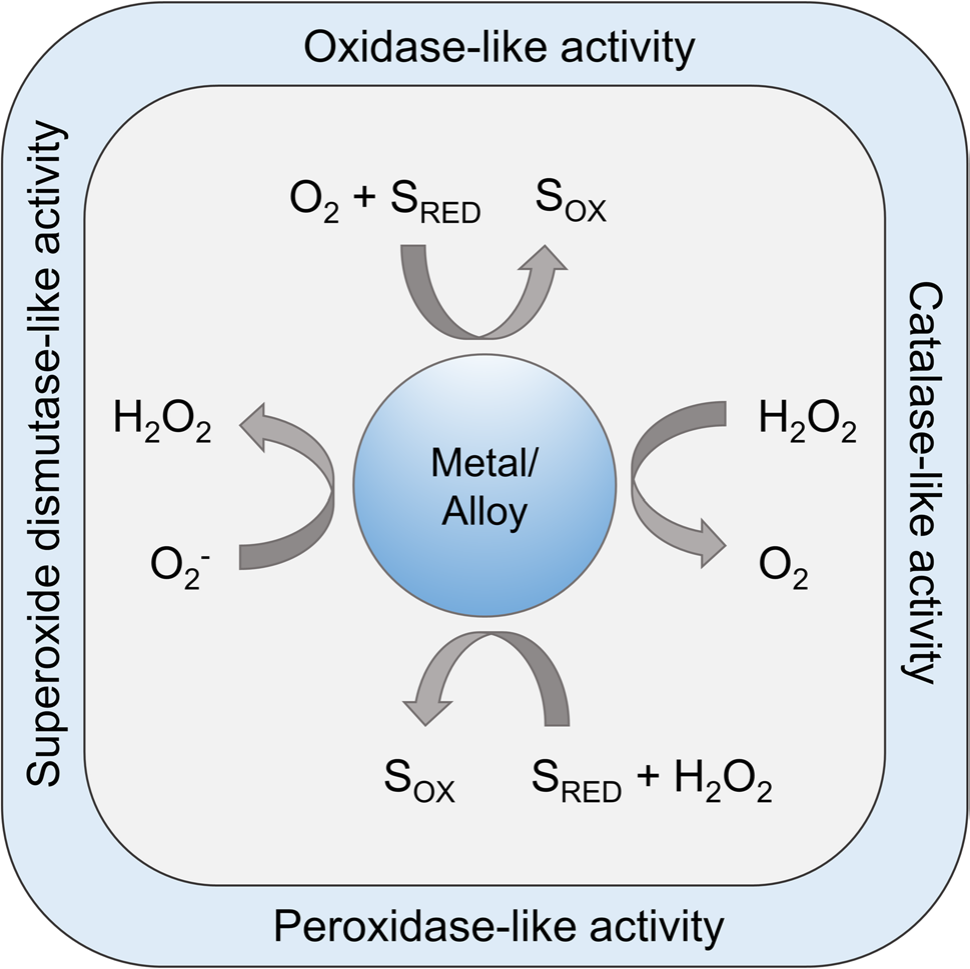
Possible enzyme-like catalytic activities of metal/metal alloy nanoparticles. Figure adapted with permission from ref. [9]; copyright © 2015, American Chemical Society

Catalytic electrochemical sensors can detect the conversion of a chemical compound, which can be directly or indirectly correlated to the analyte’s concentration in a sample. The type of catalyst used to drive the chemical reaction classifies sensors as either enzymatic or nonenzymatic. Although numerous enzymatic biosensors provide high sensitivity and selectivity, they often exhibit significant shortcomings, narrowing their application range. The most frequently encountered limitations include a short shelf-life (caused by the fragility of enzymes), stringent operating conditions (caused by the low tolerance of enzymes to the environment fluctuations), and low reproducibility and repeatability, as well as high production costs [1]. Consequently, the research focus has shifted towards nonenzymatic sensors, including devices that utilise unique characteristics of metal nanoparticles (NP) [2–6]. It has been shown that certain nanoparticles, also referred as nanozymes, can catalyse chemical reactions due to their oxidase-like, peroxidase-like, catalase-like, and/or superoxide dismutase-like activity (Fig. 1); hence, they serve as a cheaper, more robust, easier to handle, and more stable alternative to enzymes [7, 8]. Nanoparticle thermal stability is especially beneficial when designing POC devices to be used in low-resource settings with poor storage conditions, including limited access to refrigerators. The high stability of biosensors allows for their long-term storage and facilitates their delivery to off-grid communities, for example, in rural parts of sub-Saharan Africa.

The size, shape, morphological uniformity, chemical purity, dispersity, and environment of metal nanoparticles determine their catalytic properties and applicability. These parameters can be tuned by varying synthesis conditions such as temperature, type and amount of a metal precursor, type and amount of a reducing agent, and a stabiliser (capping agent) [10]. In some cases, microporous hosts can be employed to control the catalytic processes at embedded nanoparticles [11].

**Fig. 2:**
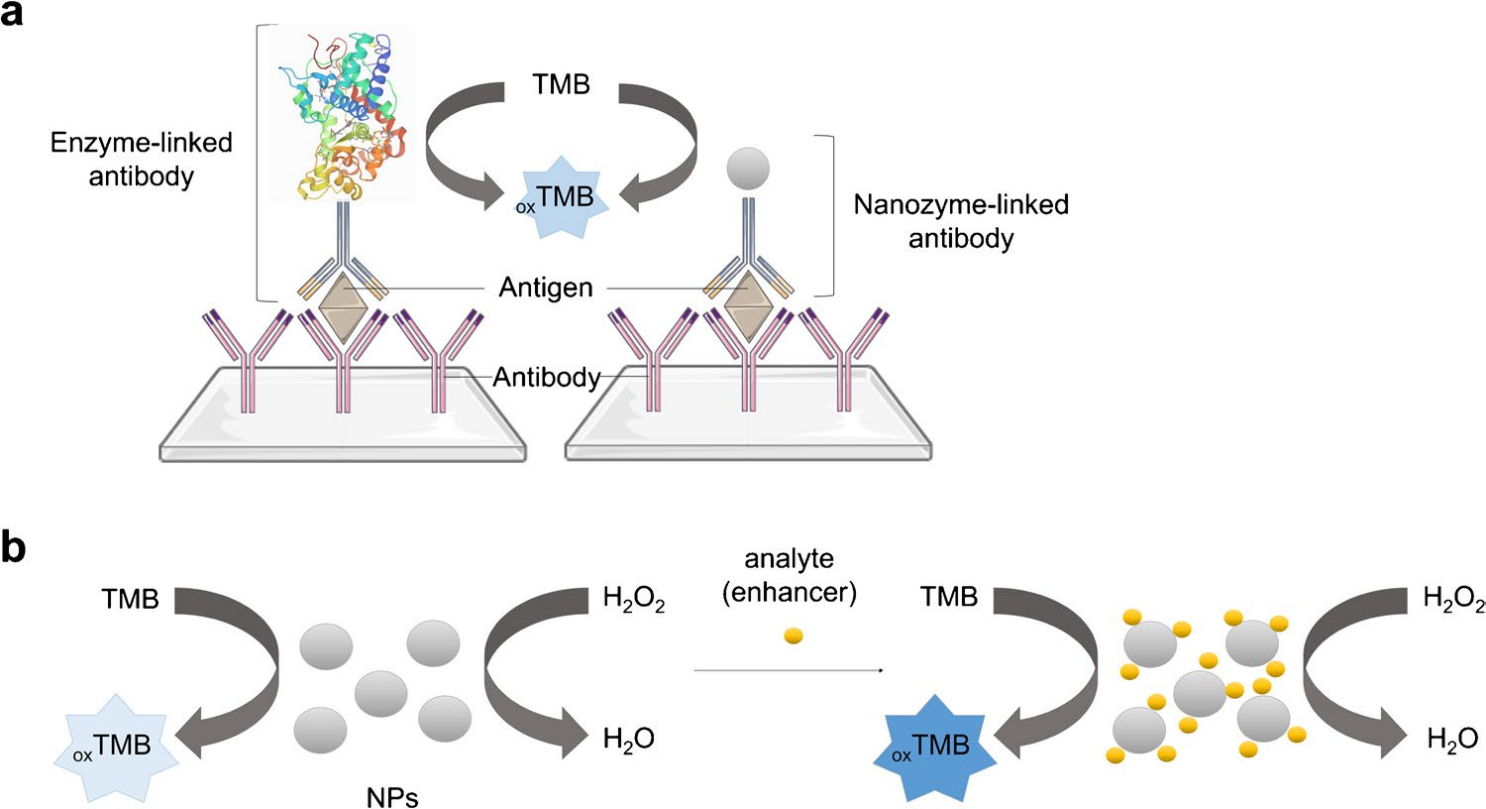
Examples of metal NP application in sensors. **a** Metal NP as a signal amplifier. Adapted from ref. [12]; used under CC BY-NC-ND 4.0 license (http://creativecommons.org/licenses/by-nc-nd/4.0/). **b** Detection of an analyte based on its modulation effect on metal NP catalytic activity. Adapted with permission from ref. [13]; copyright © 2015 Elsevier Inc

Metal nanoparticles can play different roles in sensing devices. For example, they can replace enzymes such as horseradish peroxidase (HRP) in sandwich-like assays (Fig. 2a) [12]. Immunosensors and immunoassays utilising nanoparticles in this context were reviewed by Niu et al. [12]. In other applications, metal nanoparticle activity is modulated by an analyte, allowing to correlate a catalysed reaction rate to an analyte concentration (Fig. 2b) [13]. In this case, a substrate for a nanoparticle-catalysed reaction is abundant, whereas an analyte acts either as an inhibitor or an activator (enhancer). Sensors based on this mechanism can be used for heavy metal detection and were reviewed by Unnikrishnan et al. [14]. Finally, metal nanoparticles can directly catalyse an analyte conversion, allowing for its electrochemical detection, the mechanism of which is the focus of this review.

Most reviews in the literature organise nanozymes based on their application. Zhang et al. reviewed colorimetric, fluorescence, chemiluminescent, and electrochemical nanozyme-based biosensors for food contaminant detection such as antibiotics, pesticides, and pathogens [3]. Zhang et al. described examples of nanozymes used for diagnosis (e.g. cancer and metabolic diseases) and therapeutics (cancer therapy, antioxidant, and antibacterial agents) [15]. Mahmudunnabi et al. wrote a detailed review on the recent advances in nanozyme synthesis and classification and their applications, including genosensors, immunosensors, cytosensors, and aptasensors. The authors provided an insightful and critical view on the challenges and future perspectives of nanozymes [16].

A review by Wang et al. focuses on recent advances in the field. The authors explained nanoparticle structure–function relationship and principles underlying various enzyme-like activities (e.g. oxidase, peroxidase). They discussed nanozyme applications and, finally, discussed challenges and perspectives [17]. The importance of in-depth knowledge about nanozymes’ catalytic properties and improving their selectivity was emphasised. The authors suggested that building a catalogue of nanozymes relating their structure with catalytic performance would be a useful tool to facilitate finding appropriate nanozyme with the desired function. To address this, we compiled numerous examples of metal-based nonenzymatic sensors to relate metal nanoparticle catalytic properties not only with structure but also with other factors such as operating conditions.

Zhou et al. summarised the challenges limiting the nanozyme application, including their low selectivity compared to enzymes [4]. Although numerous metal and metal oxide nanoparticle-based sensors are described in the literature, the detection mechanism often remains vague. Many nanomaterials can catalyse more than one reaction type and respond to various molecules. Hence, to design a highly selective sensor, it is crucial to understand how nanoparticle catalytic activity can be tuned, which is the aim of this review. To address this challenge, a different approach from most previous reviews was taken. Sensors are categorised by the type of metals/metal oxides rather than their application/detected analyte. Focusing on each material separately puts stress on metal catalytic activity depending on structure/size and the environmental conditions. Table 1 lists several frequently analysed molecules and depicts which metals can be used for their detection depending on pH and environment. Studying and comparing numerous examples of metal nanoparticle-based sensors can unravel crucial factors affecting the catalytic properties of nanomaterials. Reactivity at nanoparticles is affected by very fast mass transport at nanoscale and, therefore, often different from bulk reactivity, especially when hydrogen peroxide is produced [18].

This review intends to help researchers design highly sensitive nonenzymatic sensors by choosing (i) the most suitable material serving as an enzyme mimetic component and (ii) the optimal conditions embracing its catalytic activity. It focuses exclusively on the electrochemical sensors in which metal nanoparticles are directly involved in an analyte conversion. In order to provide a comprehensive view of the subject, the biosensors described are sorted by the type of metal used as an enzyme alternative. The research papers providing a detection mechanism were the primary choice for this review. The examples represent each metal in various environmental conditions that affect catalytic properties. In order to help the reader to navigate through the review, we recommend first finding a nanozyme suitable for an application of interest in Tables 2, 3, and 4 and then exploring properties and challenges in the section devoted to this metal.

**Table 1:** pH-dependent selectivity of various metal/metal alloy nanomaterials.

Analyte	pH		
	Acidic	Neutral	Alkali
Glucose (oxidase)	×	Platinum Iron	Cobalt Copper Copper/palladium Nickel Copper/nickel Nickel/manganese Palladium/gold
Dopamine (oxidase/peroxidase)	Nickel	Cobalt Copper	×
Hydrogen peroxide (catalase/peroxidase)	Silver	Silver Iron Cobalt Copper Gold Palladium Gold/palladium Platinum	Cobalt Cobalt/nickel
Superoxide anion (superoxide dismutase)	×	Copper Iron	×

Information in brackets indicates enzyme-like activity required to detect a particular analyte; × indicates that no examples were found in the literature.

**Table 2:** Overview of metal-based electrochemical glucose sensors

Metal	Electrolyte	pH	Potential [V vs Ag/AgCl]	Linear range [μM]	LOD [μM]	Sensitivity	Area [cm^2^]	Ref
Co	NaOH	13	0.58	Up to 80	0.16	122.16 μA mM^−1^ cm^−2^	N/A	[19]
Co	NaOH	13	0.59	20–250	1.5	5.65 × 10^3^ μA mM^−1^ cm^−2^	0.07	[20]
Co	NaOH	13	0.6	5–2.65 × 10^3^; 4.65 × 10^3^–13.6 × 10^3^	5	86.6 µA mM^−1^ cm^−2^	0.2	[21]
Ni	NaOH	13	0.5	0.5–20	0.0	4.25 × 10^3^ µA mM^−1^ cm^−2^	0.07	[22]
Ni	NaOH	13	0.55	0.5–9 × 10^3^	7 × 10^−3^	4.4 × 10^3^ µA mM^−1^ cm^−2^	0.07	[23]
Ni/Mn	NaOH	13	0.45	1–1 × 10^3^; 1 × 10^3^–3.5 × 10^3^	N/A	2.61 × 10^3^ µA mM^−1^ cm^−2^; 294.8 µA mM^−1^ cm^−2^	1	[24]
Co/Ni	NaOH	13	0.5	0.5–590	0.38	2.52 × 10^3^ µA mM^−1^ cm^−2^	N/A	[25]
Co/Ni	KOH	13	0.54	1–4.55 × 10^3^	0.18	3.76 × 10^3^ μA mM^−1^ cm^−2^	0.07	[26]
Co/Ni	KOH	14	0.43	0.1 × 10^4^–2 × 10^4^	N/A	2.6 × 10^3^ μA mM^−1^ cm^−2^	~ 0.5	[27]
Co/Ni	NaOH	13	0.5	5–1.09 × 10^4^	0.39	2.07 × 10^3^ μA mM^−1^ cm^−2^	~ 0.01	[28]
Co/Ni	NaOH	13	0.55	1–1.13 × 10^3^	0.64	1.28 × 10^4^ μA mM^−1^ cm^−2^	6	[29]
Cu	NaOH	13	0.64	20–2 × 10^4^	0.06	472 µA mM^−1^ cm^−2^	0.28	[30]
Cu	NaOH	13	0.35	1–1.88 × 10^4^	0.3	2.22 × 10^3^ µA mM^−1^ cm^−2^	1	[31]
Cu	NaOH + KCl	N/A	0.45	0.1–400	0.04	830 µA mM^−1^ cm^−2^	0.16	[32]
Cu/Co	NaOH	13	0.55	1–500	0.38	5.4 × 10^6^ µA mM^−1^ cm^−2^	1	[33]
Cu/Pd	NaOH	13	0.65	0.5–2.6 × 10^3^	0.1	1.02 × 10^3^ μA mM^−1^	1	[34]
Cu	KOH N_2_-saturated	13	0.6	300–3.3 × 10^3^	3.3	285 μA mM^−1^ cm^−2^	0.07	[35]
Fe	PBS	7.4	0.44	15–3 × 10^3^	7.8	12.13 µA mM^−1^	N/A	[36]
Au/Pd	NaOH	13	− 0.06	500–2 × 10^4^	400	738 µA mM^−1^	0.07	[37]
Pt	PBS N_2_-saturated	7.4	0.6	300–1 × 10^4^; 1 × 10^4^–5 × 10^4^	100	0.19 μA mM^−1^; 0.07 μA mM^−1^	0.07	[38]

*LOD*, limit of detection; *PBS*, phosphate buffer saline; *N/A*, data not available; *V* indicates approximate working potential; *Area* specifies geometrical area of electrode.

**Table 3:** Overview of metal-based electrochemical hydrogen peroxide sensors

Metal	Electrolyte	pH	Potential [V vs Ag/AgCl]	Linear range [μM]	LOD [μM]	Sensitivity	Area [cm^2^]	Ref
Co	PBS	7.4	− 0.2	5–1.1 × 10^4^	1	87.4 μA mM^−1^ cm^−2^	0.2	[21]
Co	NaH_2_PO_4_-NaOH buffer	10	− 0.74	10–4 × 10^3^	4.4	N/A	0.07	[39]
Co/Ni	NaOH	13	0.5	1–510	0.14	59.7 μA mM^−1^	~ 0.01	[28]
Cu	PB N_2_-saturated	7.4	− 0.4	300–7.8 × 10^3^	20.8	N/A	0.07	[35]
Fe	PBS	7	− 0.3	0.5–3 × 10^3^	0.18	22.27 µA mM^−1^ cm^−2^	N/A	[40]
Fe	PBS	7	− 0.4	200–2 × 10^3^	10	1.82 μA mM^−1^	2.25	[41]
Fe	PB	7.5	− 0.2	20–1 × 10^3^	2	1.38 µA mM^−1^	N/A	[42]
Au	PBS	7	− 0.45	10–2.73 × 10^3^	6.35	13.2 µA mM^−1^	0.13	[43]
Pd	PBS	7	− 0.2	53–4.4 × 10^3^	13.3	368 µA mM^−1^ cm^−2^	0.05	[44]
Pd	PB + KCl	7	− 0.1	10–1 × 10^4^; 250–5 × 10^4^	3	79.28 µA mM^−1^ cm^−2^	0.16	[45]
Au/Pd	PB N_2_-saturated	7.4	− 0.6	5–1.15 × 10^4^	1	186.86 µA mM^−1^ cm^−2^	2	[46]
Au/Pd	PBS	7.4	− 0.07	0.8–1 × 10^4^	0.16	184.9 µA mM^−1^ cm^−2^	0.07	[37]
Pt	PBS N_2_-saturated	7.4	− 0.1	0.3–2.34 × 10^3^	0.1	18.2 μA mM^−1^	0.07	[38]
Ag	acetate buffer deareated	3	− 0.12	62.34–2.4 × 10^3^	6.25	120 μA mM^−1^	0.07	[47]
Ag	PBS	7.4	0.6	50–2.5 × 10^3^	1	48.74 µA mM^−1^ cm^−2^	0.2	[48]
Ag	PBS	7	− 0.2	50–1.7 × 10^4^	0.5	1.42 µA mM^−1^	0.07	[49]

*LOD* Limit Of Detection; *PB* Phosphate Buffer; *PBS* Phosphate Buffer Saline; *N/A* data not available; *V* indicates approximate working potential; *Area* specifies geometrical area of electrode.

**Table 4:** Overview of metal-based electrochemical sensors for various analytes

Metal	Electrolyte	pH	Potential [V vs Ag/AgCl]	Linear range [μM]	LOD [μM]	Sensitivity	Area [cm^2^]	Ref
Dopamine								
Co	PB	7	0.29	10–240	0.02	8 × 10^4^ µA mM^−1^ cm^−2^	N/A	[50]
Ni	KCl N_2_-saturated	5	0.65	40–1.08 × 10^3^	0.26	104.17 µA mM^−1^	0.2	[51]
Ni	PB	6	0.24	0.01–200	1 × 10^−3^	630.4 μA mM^−1^	N/A	[52]
Cu	PB	7.4	− 0.05	Up to 30	5	N/A	0.2	[53]
Cu	PB	7.2	− 0.01	30–320	8	503 µA mM^−1^	0.07	[54]
Cu	BRB + H_2_O_2_	7	0.03	5–130	0.52	21.2 μA mM^−1^ cm^−2^	0.2	[55]
Cu	PB + H_2_O_2_	7	− 0.01	35–240	8	2.02 μA mM^−1^ cm^−2^	0.03	[56]
Catechol								
Cu	PB	7.4	− 0.05	Up to 200	3	N/A	0.2	[53]
Cu	NaOH	13	0.54	20–1 × 10^4^	7 × 10^−3^	7.12 × 10^5^ µA mM^−1^ cm^−2^	0.28	[57]
Cu	PB + H_2_O_2_	7	− 0.01	100–250	N/A	0.82 μA mM^−1^ cm^−2^	0.03	[56]
Fe	Succinate buffer + H_2_O_2_	4	0.09	0.6–6	0.35	61 μA mM^−1^ cm^−2^	0.07	[58]
Oxalic acid								
Co	KCl N_2_-saturated	7	0.77	38.9–460	0.58	451.18 µA mM^−1^	0.2	[59]
L-dopa								
Cu	PB + H_2_O_2_	7	− 0.01	30–190	N/A	0.34 μA mM^−1^ cm^−2^	0.03	[56]
4-Aminophenol								
Cu	BRB + H_2_O_2_	7	− 0.03	5–230	2 × 10^−3^	123 μA mM^−1^ cm^−2^	0.2	[60]
Hydrazine								
Au	PBS N_2_-saturated	7.4	0.19	5–645; 645–3.34 × 10^3^	1.1	59.09 µA mM^−1^; 36.05 µA mM^−1^	0.07	[61]
Pd	PB N_2_-saturated	7	0.12	0.05–56.45; 56.45–406.45	7 × 10^−3^	343.91 μA mM^−1^; 186.52 μA mM^−1^	0.2	[62]
Cu	PBS	5	0.25	50–800	4.3	8.5 µA mM^−1^	0.11	[63]
Ni	NaOH	13	− 0.1	N/A	N/A	N/A	0.07	[64]
Superoxide anion								
Cu	PB	7.4	0.25	3.1–326	0.25	70 µA mM^−1^ cm^−2^	0.05	[65]
Fe	PB	7.4	0.54	0.01–10	0.01	9.6 × 10^3^ µA mM^−1^ cm^−2^	N/A	[66]
NO								
Fe	PB	7.4	0.79	0.02–9	7 × 10^−3^	290 μA mM^−1^	N/A	[67]
Au	PB	4.4	0.98	0.25–2.25	2.4 × 10^−4^	1.31 × 10^4^ µA mM^−1^	N/A	[68]
Ascorbic acid								
Ni	KCl N_2_-saturated	5	0.39	90–2.11 × 10^3^	0.45	53.02 µA mM^−1^	0.2	[51]
Calcium								
Co/Ni	Tris buffer + H_2_O_2_	N/A	− 0.6	30–460	4.45	284.54 μA mM^−1^ cm^−2^	N/A	[25]
Lactic acid								
Co/Ni	KOH	14	0.54	5 × 10^3^–5 × 10^4^	N/A	430 μA mM^−1^ cm^−2^	~ 0.5	[27]

*LOD*, limit of detection; *PB*, phosphate buffer; *PBS*, phosphate buffer saline; *BRB*, Britton–Robinson buffer; *RE*, reference electrode; *N/A*, data not available; *V* indicates approximate working potential; *Area* specifies geometrical area of electrode.

## Cobalt oxide

Cobalt oxide (Co_3_O_4_) is a p-type semi-conductive mixed valency compound consisting of Co^2+^ and Co^3+^ ions; thus, its formula can also be written as CoO·Co_2_O_3_. It has attracted considerable attention due to its catalytic properties and high stability in alkaline media. For instance, cobalt oxide has been extensively studied for glucose detection, performed at pH 13–14 in either NaOH or KOH solution [19–21]. Cyclic voltammetry (CV) analysis of a cobalt oxide-modified electrode shows two pairs of redox peaks attributed to the conversion of Co_3_O_4_ to CoOOH, followed by its oxidation to CoO_2_, and the reverse reactions [20]. Glucose oxidation is a two-electron process in which gluconolactone is produced. Upon adding glucose to the supporting electrolyte, the current of all four peaks increases, suggesting that cobalt oxide is involved in glucose oxidation.

**Fig. 3:**
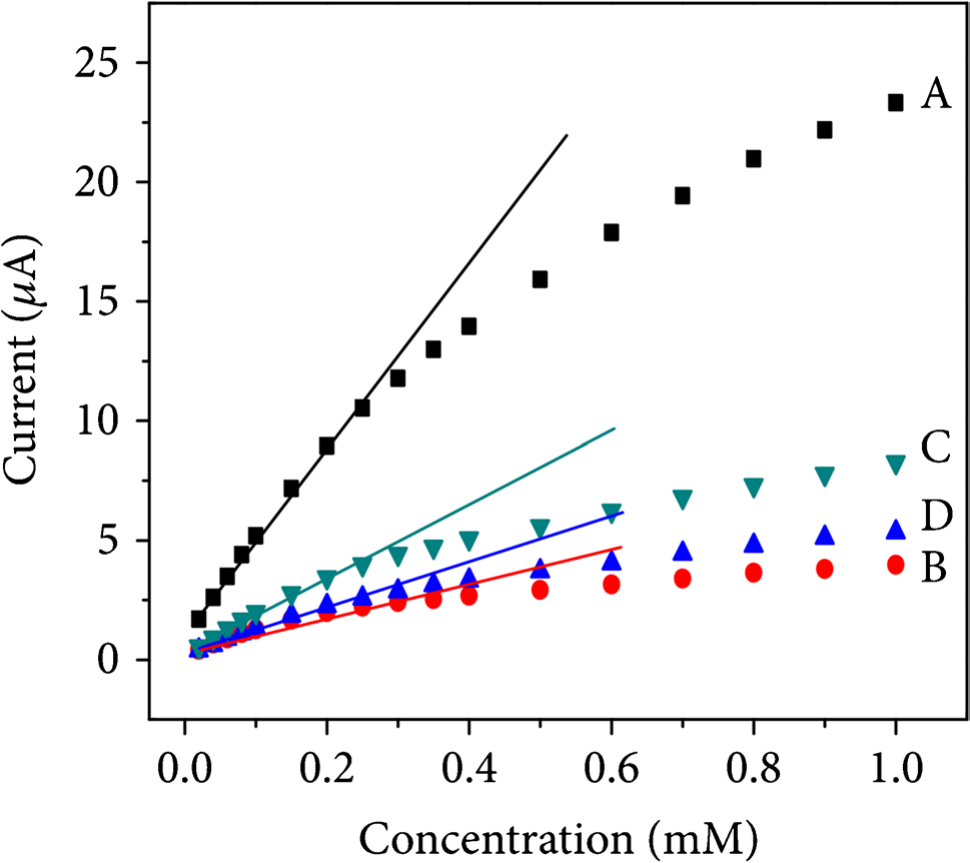
Peak current as a function of glucose concentration obtained using various Co_3_O_4_ nanostructures **A** nanourchins, **B** nanowires, **C** nanoflowers, and **D** nanoplates. Figure adapted from ref. [20]; copyright © 2019 Jiankang Luo et al. Used under CC BY license 4.0 (http://creativecommons.org/licenses/by/4.0/)

Luo et al. focused their research on demonstrating that the catalytic activity of different Co_3_O_4_ nanostructures depends on their shape [20]. To do so, they performed a shape-controlled Co_3_O_4_ nanostructure synthesis resulting in nanourchins, nanowires, nanoflowers, and nanoplates. Nanourchins provided the best sensor characteristics when used for a glassy carbon electrode (GCE) modification. The response of the nanourchin-modified sensor is almost 6 times higher than that of the nanowire-modified sensor. The authors attribute the improvement to the nearly 7 times higher surface area and more accessible active sites for nanourchins compared to nanowires (Fig. 3). This research highlights the impact of nanomaterial morphology on sensing performance.

**Fig. 4:**
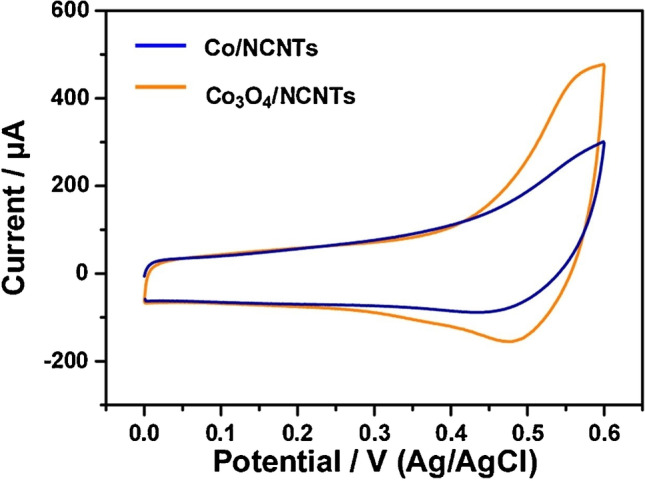
Cyclic voltammograms of Co/NCNTs and Co3O4/NCNTs electrodes in 0.1 M NaOH without the presence or with the absence of mM glucose. Figure reproduced with permission from ref. [21]; copyright 2019 Elsevier B.V

Qin et al. compared sensing performance of cobalt oxide-based and metallic cobalt-based sensors, showing the former’s advantage, supposably due to the energetically favoured glucose oxidation by the high valence cobalt (Fig. 4) [21].

Cobalt oxide finds an application in other important molecule detections, such as hydrogen peroxide. The biosensor described above, designed by Qin et al., is a good example of a bifunctional sensing device. By changing environmental conditions, substituting NaOH solution with a phosphate buffer saline (PBS) at pH 7.4, the authors quenched Co_3_O_4_ nanoparticle affinity to glucose and enhanced their affinity to hydrogen peroxide.

Mu et al. expanded the mechanism study of a cobalt oxide-mediated decomposition of hydrogen peroxide [39]. In an aqueous solution, hydrogen peroxide reacts with hydroxide ions (a). Hydroperoxo anions (OOH^−^) produced in this reaction, together with cobalt, take part in a two-step ·O_2_H formation (b, c). Co_3_O_4_ nanoparticles adsorb the analyte (H_2_O_2_) onto their surface, leading to a Co(II) activation and a formation of hydroxyl radicals (·OH) (d), which interacting further with ·O_2_H produce water and oxygen molecules (e):

- H_2_O_2_ + OH^−^ ↔ OOH^−^ + H_2_O
- Co(III) + OOH^−^ → Co(II)·OOH
- Co(II)·OOH → Co(II) + ·O_2_H
- Co(II) + H_2_O_2_ → Co(III) + ·OH + OH^−^
·OH + ·O_2_H → H_2_O + O_2_

**Fig. 5:**
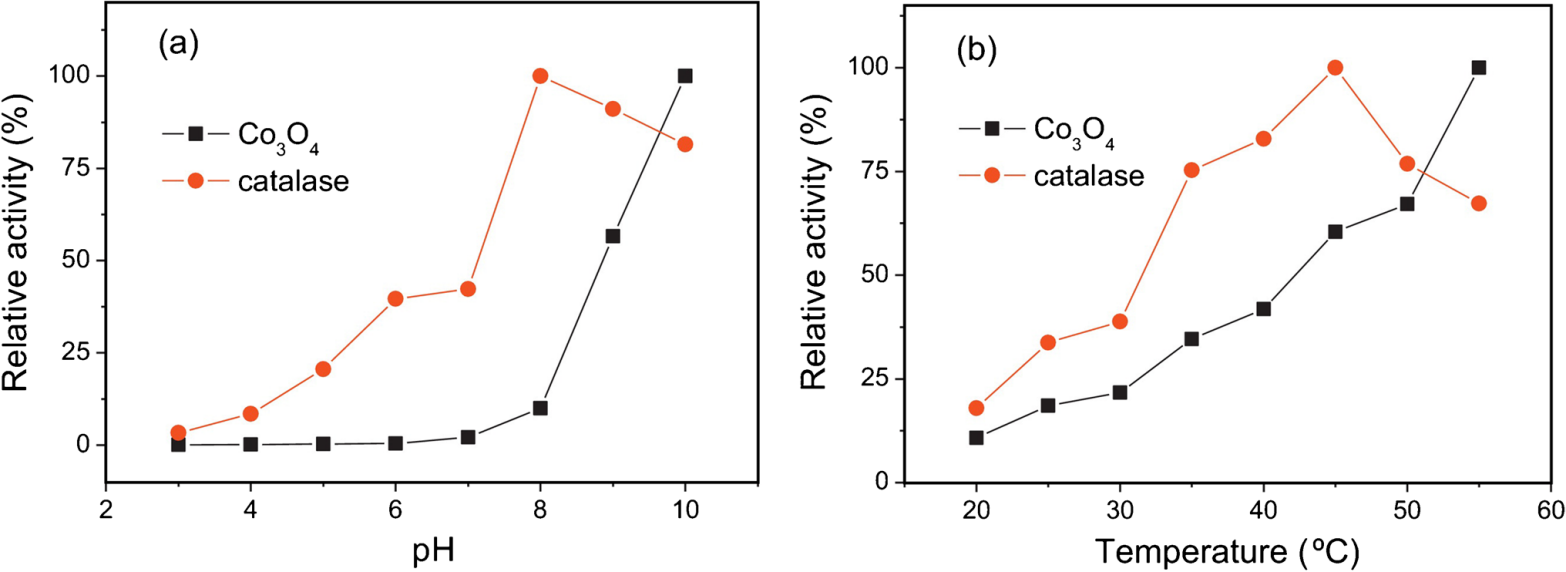
PH (**a**) and temperature (**b**) effect on the catalase-like activity of Co_3_O_4_. Co3O4, 20 μg mL^−1^ in 100 mM NaH_2_PO_4_-NaOH buffer (pH 10); catalase, 0.5 U mL^−1^ in 100 mM sodium phosphate buffer. Figure reproduced with permission from ref. [39]; copyright © 2013 Elsevier B.V

The biosensor studied by Mu et al. was prepared by drop-casting Co_3_O_4_ nanoparticles onto a GCE. According to the optimisation study, the cobalt oxide reaches its maximum catalase activity at pH 10 (pH range from 3 to 10 was tested), which can be explained by the higher concentration of hydroperoxo anions in an alkaline solution required to drive the reaction (b) and (c). Furthermore, the temperature effect on the sensor performance was tested. The catalase activity was shown to increase proportionally with a temperature increase from 20 to 55 °C. It is generally known that particles’ motion speed is boosted at higher temperatures enhancing the frequency of intermolecular interactions. This optimisation study underlines the advantage of using metals over enzymes for sensing purposes due to the fragility of proteins to extreme environmental conditions (Fig. 5). Considering the optimum pH for the presented sensor was shown to be pH 10, it would be beneficial to perform an interference study taking into account that the previous examples show the affinity of Co_3_O_4_ nanoparticles to glucose in alkaline media.

An oxidase-like cobalt activity was utilised to detect dopamine [50] and oxalic acid [59]. In both cases, for a disc sensor construction, the authors used a mesoporous carbon ceramic electrode (CCE) based on a hybrid of silica (SiO_2_) and graphite (C). The design provided a versatile, mechanically stable, and resistant to harsh environment support with tunable porosity for anchoring electroactive species. The dopamine biosensor was further functionalised by the in situ synthesis of cobalt oxide nanoparticles. The electrochemical studies showed that a SiO_2_/C/Co_3_O_4_ electrode catalyses dopamine oxidation, which results in the anodic peak at the potential + 0.25 V vs SCE, in contrary to SiO_2_/C and SiO_2_/C/Co electrodes, which highlights the significance of a mixed valency of cobalt oxide in the oxidation process. The sensor response to interferents, such as ascorbic acid and uric acid, was minimal.

On the other hand, the oxalic acid (H_2_C_2_O_4_) biosensor was prepared by functionalisation of SiO_2_/C matrix with in situ synthesised cobalt (II) phthalocyanine (CoPc). Metallophthalocyanines (MPc) are aromatic macrocyclic synthetic compounds with a metal cation coordinated in the centre [69]. The structure is closely related to that of heme-porphyrin ligands complexed with an iron molecule — a natural component in many enzymes, which attracted attention to the catalytic properties of MPc [70]. It has been shown that MPc can be easily modulated by incorporating different metal ions in its centre and various substituents [71]. Rahim et al. performed a chronoamperometric oxalic acid determination under a nitrogen atmosphere in a KCl solution at a neutral pH (changing pH from 2 to 9 showed insignificant changes in response). Oxidation of oxalic acid is likely mediated via Co(II) → Co(III) → Co(II) regeneration process.

## Nickel and nickel oxide

Immersing a nickel-functionalised electrode in alkali media leads to Ni(III) oxyhydroxide formation. Its ability to catalyse organic compound oxidations has been utilised for amperometric glucose detection [22–24].

Singer et al. prepared NiO nanostructures on Ni foil by a glancing angle deposition (GLAD) technique, which provided high control over a fabricated surface morphology. Zhang et al. functionalised a GCE with N-doped carbon (NC) and reduced graphene oxide (rGO) as supporting material for NiO nanoparticles to boost their conductivity, resulting in NiO-NC@rGO/GCE microspheres [22]. Amperometric glucose determination can be performed at slightly lower working potential (+ 0.5 V vs Ag/AgCl) compared to the sensor described by Singer et al. (+ 0.55 V vs Ag/AgCl), which can be explained by the enhanced electron transfer properties. Both sensors perform glucose determination with negligible uric acid, dopamine, and ascorbic acid interference. Singer et al. expanded the list of possible interferants with serotonin whereas Zhang et al. with KCl, neither of which affected the sensors’ performance.

Nickel and manganese synergistic activity in alkali media was employed by Dong et al. in a binary glucose biosensor based on NiMn_2_O_4_ nanosheets anchored in rGO [24]. Both metals participate in an electrooxidation of glucose occurs via oxyhydroxides. The sensor selectivity was examined with uric acid, urea, dopamine, and ascorbic acid, which did not show any interference with glucose detection.

Another nickel application was presented by Vikraman and Park, who designed a NiO-functionalised GCE modified with a Nafion® membrane for hydrazine oxidation at alkali pH [64]. Although the work does not focus on the sensor features (such as linear range and sensitivity) nor the reaction mechanism, it provides an insight into the nanostructures’ size and shape-modulated catalytic activity. Both characteristics were controlled by the type of reducing agent used in the synthesis process. Results are presented in Table 2. Amongst nanodots, nanorods, nanocubes, and nanopellet, the latter exhibits the highest electrocatalytic activity resulting in the lowest oxidation peak potential for hydrazine (953 mA g^−1^ and 503 mA g^−1^ for nanopellet and nanocubes, respectively). The authors explained this by a high active surface area to volume ratio, the existence of active crystalline facets, and a charge density at the apex of nanopellets induced by their curvature. Furthermore, it has been demonstrated that capping nanostructures with a silica shell (SiO_2_) decreases their catalytic activity by 4 times, probably by limiting the accessibility of nanomaterial active sites.

**Fig. 6:**
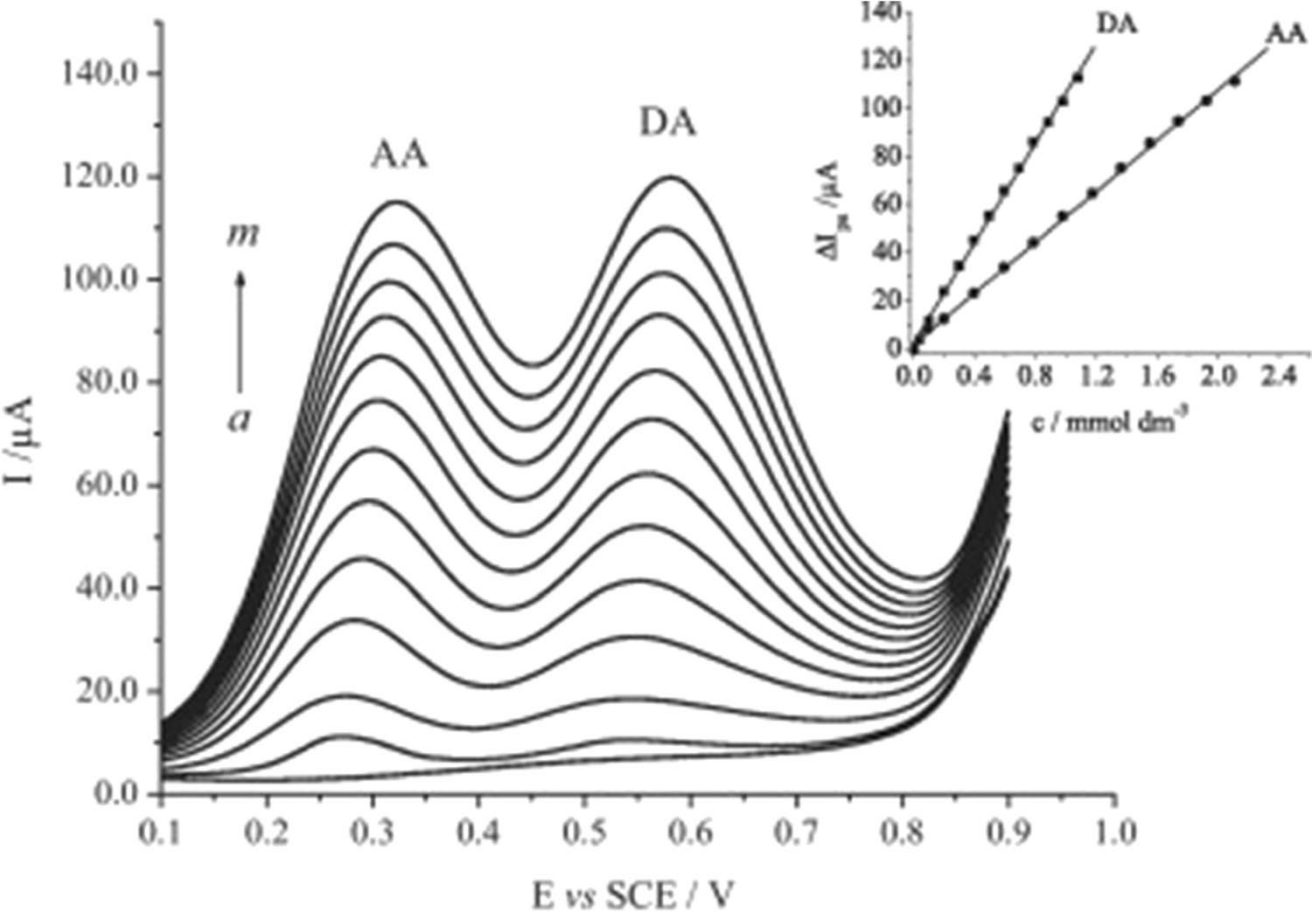
Differential pulse voltammogram response of SGN/NiPc electrode to increasing concentrations of ascorbic acid (AA) and dopamine (DA) in 0.5 M KCl solution (pH 5.0). DA, (a) 40.0 µM – (m) 1.08 mM. AA, (a) 90.0 µM – (m) 2.11 mM. Inset: respective calibration curves for AA and DA. Figure reproduced with permission from ref. [51]; copyright © 2012 Elsevier Ltd

Oxidative properties of nickel can be utilised for dopamine and ascorbic acid detection in a slightly acidic media (pH 5–6) and under a nitrogen atmosphere [51, 52]. Barros et al. constructed a biosensor based on nickel phthalocyanine (NiPc) immobilised on a mesoporous SiO_2_/C–modified disc CCE, similar to an oxalic acid sensor based on CoPc designed by Rahim et al. [51, 59]. The sensor was applied to simultaneously determine dopamine and ascorbic acid in a KCl solution (Fig. 6). In the optimum conditions (pH 5), a complete separation of two well-defined oxidation peaks is obtained at the potentials + 0.605 V and + 0.345 V vs SCE, respectively. An interference study with other co-existing in body fluids molecules would be beneficial to determine the selectivity of the device.

## Cobalt–nickel oxides

A combination of cobalt and nickel (NiCo_2_O_4_) for sensing purposes has been shown to increase the catalytic activity of sensors due to the synergistic effect of the metals, which found an application in glucose detection [25–29]. All devices perform measurements in alkali conditions (pH 13–14).

Lu et al. showed that their in-house designed biosensor can detect hydrogen peroxide and glucose. In both cases, oxidation occurs in the same conditions (alkali pH), and an oxidation peak appears at the same potential (+ 0.5 V vs Ag/AgCl) [28]. Therefore, in the presence of hydrogen peroxide and glucose in a sample, both analytes would contribute to the readout. On the other hand, dopamine, uric acid, and ascorbic acid do not influence the sensor response, likely due to their electrostatic repulsion from the electrode surface by a Nafion® film.

A sensor proposed by Elakkiya et al. was tested for both glucose and lactic acid determination, to which response occurs at a relatively close potential (0.43 and 0.54 V vs Ag/AgCl, respectively). Therefore, without a sufficient separation of the peaks, the interference of one on another should be considered when analysing samples containing both analytes.

## Copper and copper oxide

**Fig. 7:**
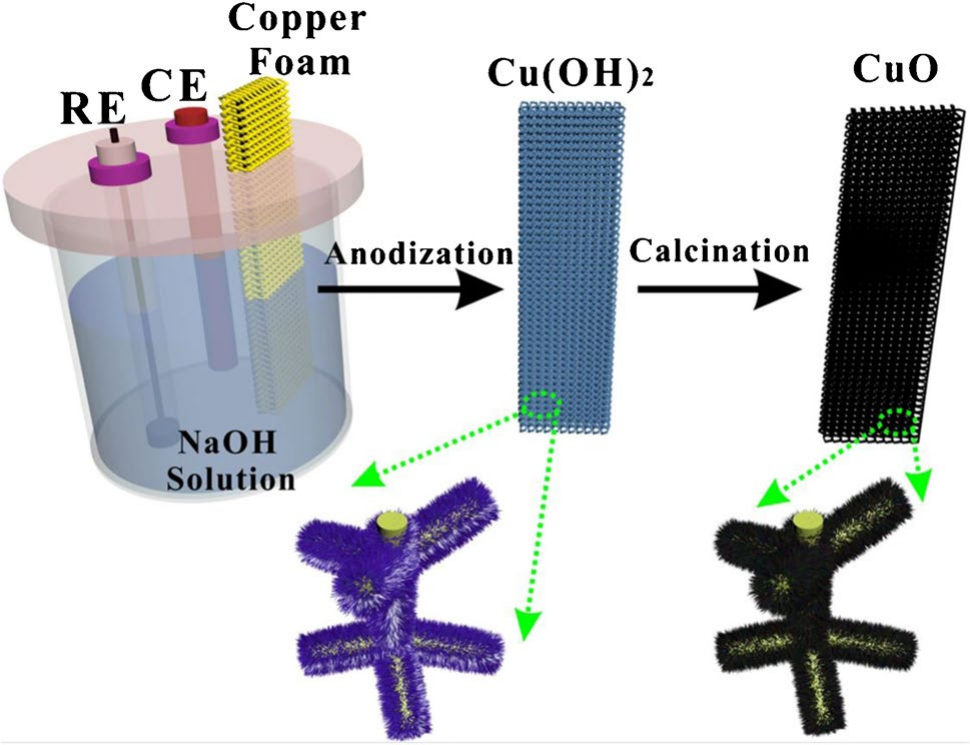
A schematic illustration of CuO NW/CFE preparation. Figure reproduced from ref. [31]; copyright © 2021 Springer Nature Limited. Used under CC BY license 4.0 (http://creativecommons.org/licenses/by/4.0/)

Copper nanoparticles have a broad application in sensing molecules such as glucose, hydrazine, hydrogen peroxide, superoxide ion, dopamine, catechol, and other phenolic compounds. In contrast to copper oxide, metallic copper is prone to poisoning by chloride ions, which reduces its electrocatalytic activity. Hence, an oxide form of copper is preferred due to its higher stability. At alkaline pH, copper oxide is converted to copper oxyhydroxide, which undergoes a reverse reaction in the presence of glucose [30]. Similarly, catalysis by metallic copper is also based on a Cu(II)/Cu(III) redox couple. The produced gluconolactone can be further hydrolysed to gluconic acid. A biosensor designed by Li et al. consists of a copper oxide-modified electrode prepared by anodisation and calcination of a copper foam electrode (CFE) (Fig. 7) [31]. A direct electron transfer and a large active surface area of the electrode provide desired sensing characteristics and a low oxidation potential of + 0.35 V vs Ag/AgCl. In comparison, most copper/copper oxide-based glucose sensors exhibit an anodic peak at a potential between + 0.45 V and + 0.65 V vs Ag/AgCl.

A sensor prepared by Ayaz et al. performs glucose measurements in 0.1 M NaOH solution with an addition of 0.1 M KCl. The device showed a negligible interference of citric acid, L-glutamic acid, sucrose, lactose, maltose, galactose, and salicylic acid; however, the response is significantly affected by dopamine, L-ascorbic, acid, and uric acid [32]. It seems that it is crucial to maintain an alkali pH to ensure the selectivity of a copper-based glucose sensor; hence, 0.1 M NaOH or 0.1 M KOH are the primary choices of a supporting electrolyte. Thus, an increased pH of the electrolyte used by Ayaz et al. may have affected their sensor’s selectivity.

**Fig. 8:**
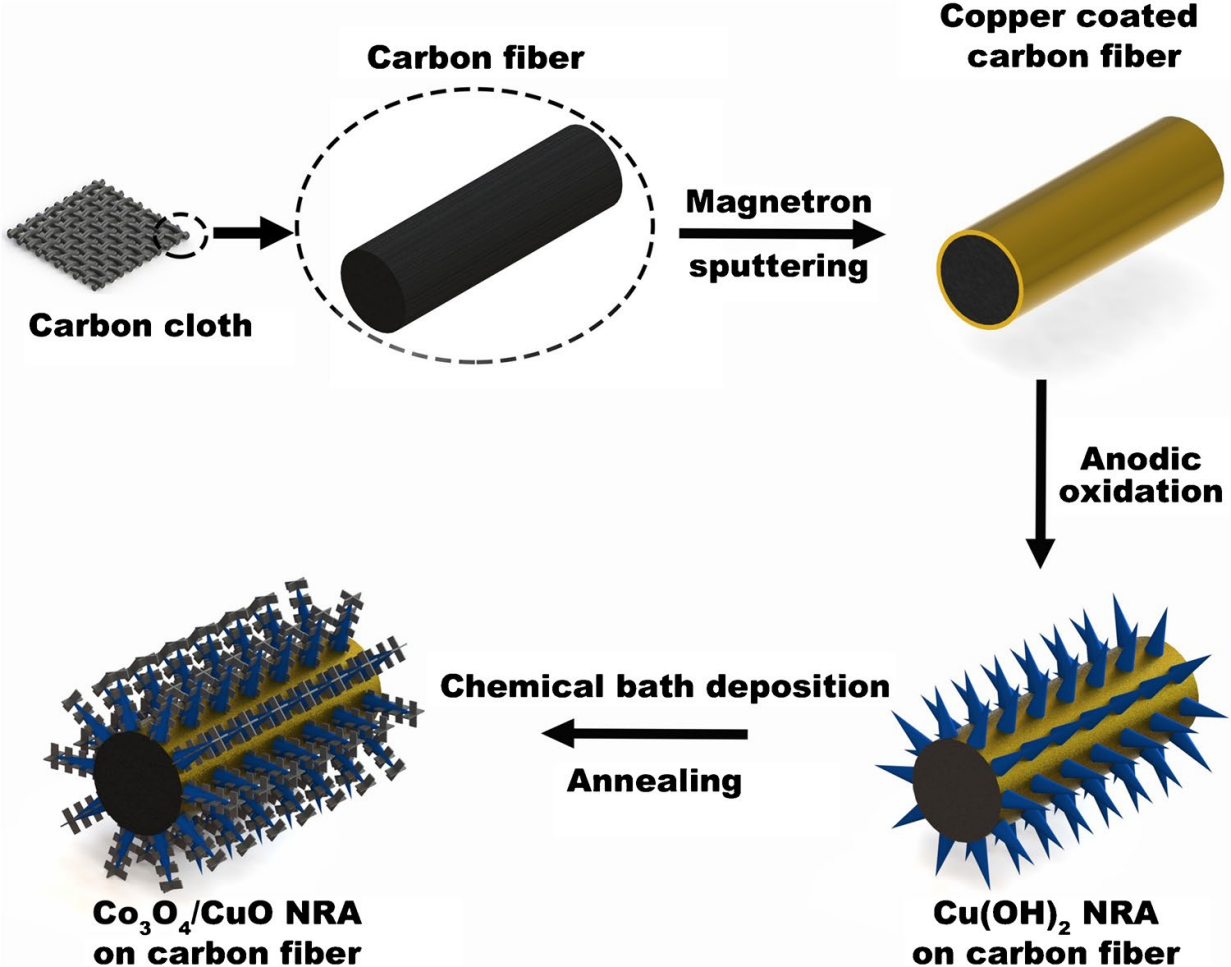
Schematic illustration of Co_3_O_4_/CuO nanorod array on carbon cloth electrode (NRA CCE) preparation. Figure reproduced with permission from ref. [33]; copyright © 2019 Elsevier B.V

**Fig. 9:**
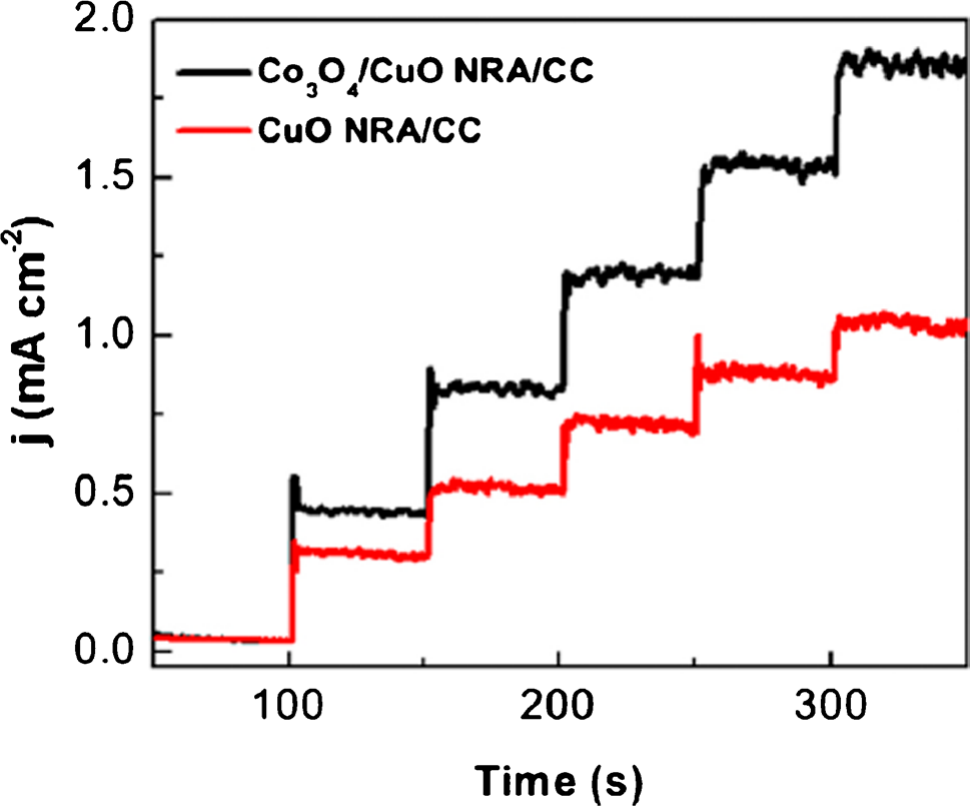
Amperometric responses of the CuO NRA/CCE (red) and Co_3_O_4_/CuO NRA/CCE (black) to the gradual addition of 50 μM glucose recorded at + 0.55 V vs Ag/AgCl. Figure reproduced with permission from ref. [33]; copyright © 2019 Elsevier B.V

Synergistic activity of copper and cobalt, as well as copper and palladium, was also applied in the development of a glucose sensor (Figs. 8 and 9) [33, 34].

Although copper retains an oxidase-like activity in neutral or slightly acidic conditions, its affinity shifts towards different molecules. In a sensor presented by Heydari et al., copper nanostructures catalyse the oxidation of hydrazine, a proton-facilitated process [63]. An optimisation study revealed that the highest anodic peak current occurs at pH 5 of 0.1 M PBS. The interference of glucose, sucrose, fructose, lactose, hydrogen peroxide, and various cations and anions is insignificant.

An enzyme-like activity of copper was studied in the detection of hydrogen peroxide. A copper (I) oxide nanocube-based biosensor designed by Liu et al. showed the ability to detect either glucose or hydrogen peroxide, depending on the sensing conditions [35]. As in other sensors, glucose detection requires an alkali pH (N_2_-saturated 0.1 M KOH), whereas hydrogen peroxide sensing is performed at pH 7.4 (N_2_-saturated 0.1 M PBS) and is facilitated by the conversion of CuO/Cu_2_O.

Dashtestani et al. showed that at pH 7.4, copper (II) complex with cysteine can serve as a superoxide dismutase mimic [65]. The most common eukaryotic SODs contain a copper and a zinc atom in their active site, with the former one playing a vital role in a superoxide anion (O_2_^●–^) scavenging.

In biosensors designed by Dashtestani et al., copper is coordinated with cysteine (Cu-Cys), which amine groups NH_3_^+^ act as cavities for superoxide ions capture. The response was further increased by attaching a Cu-Cys complex onto gold nanoparticles, which adsorb O_2_^●–^ and take part in and facilitate an electron transfer between Cu-Cys and an electrode. The sensor’s response is not affected by the presence of DMSO, citric acid, uric acid, or hydrogen peroxide.

Other molecules that can be detected at neutral pH using copper as an oxidase mimetic are phenolic compounds such as dopamine. The active site of tyrosinase, an enzyme that catalyses phenol’s oxidation, contains dinuclear copper. In order to oxidise a substrate, copper needs to be converted into a stable oxy form, either from Cu_2_^2+^ in the presence of oxygen or Cu_2_^4+^ in the presence of hydrogen peroxide [56].

**Fig. 10:**
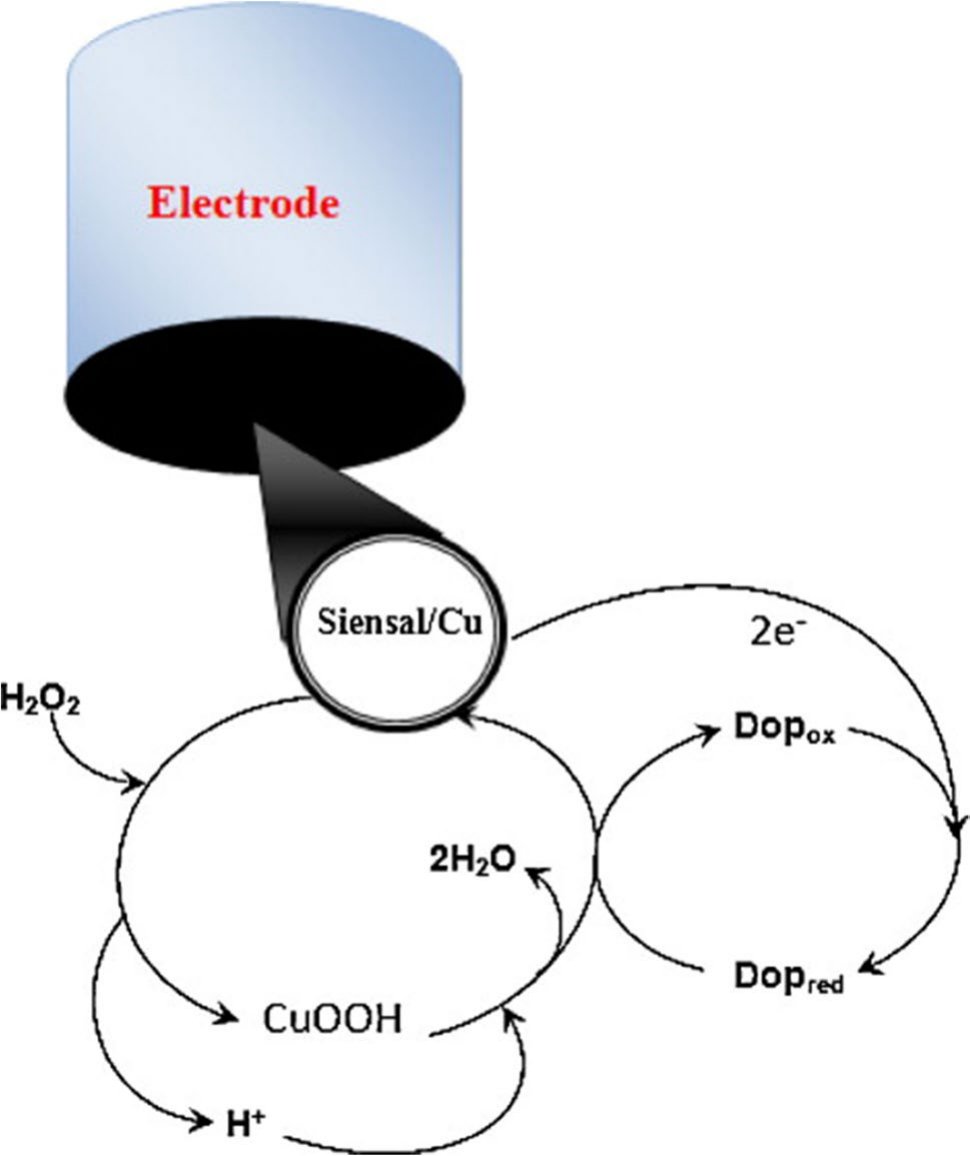
Proposed mechanism of DMS/(*ensal*)_2_Cu/C-mediated dopamine oxidation. DMS/(*ensal*)_2_Cu/C, disordered mesoporous silica modified with *ensal* organic groups mixed with graphite powder. Figure reproduced with permission from ref. [55]; copyright © 2012 Elsevier B.V

Del Pilar Taboada et al. and Dos Santos et al. presented sensors that detect dopamine at pH 7 in a buffer containing hydrogen peroxide [55, 56]. The addition of hydrogen peroxide is crucial to convert copper to its active form (CuOOH) that oxidises dopamine, which then undergoes reduction at an electrode surface (Fig. 10). The cathodic peak attributed to this reverse process increases upon the addition of the analyte. In the work of Del Pilar et al., it was shown that the sensor responds not only to dopamine, but also to catechol, L-dopa, 4-aminophenol, hydroquinone, and p-phenylenediamine. Interestingly, a copper phthalocyanine-based 4-aminophenol sensor proposed by Rahim et al. can differentiate between the analyte and other phenolic compounds such as 2-aminophenol, hydroquinone, catechol, cysteine, and resorcinol [60]. First, in the presence of hydrogen peroxide, copper phthalocyanine is oxidised, which in turn oxidises 4-aminophenol. Finally, the analyte is reduced back to its initial form at the electrode, resulting in the current increase.

**Fig. 11:**
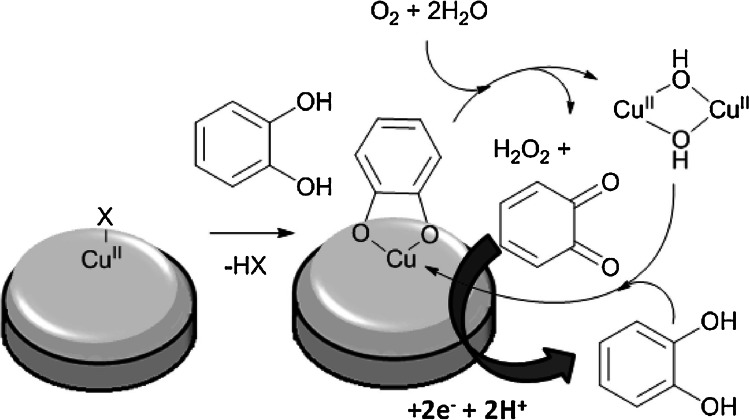
Formation of quinone at the [Cu^II^(L)Cl]Cl-modified GC electrode. Figure reproduced with permission from ref. [54]; copyright © 2016 Elsevier Ltd

On the other hand, Zen et al. and Boulkroune et al. presented dopamine sensors that do not require the addition of hydrogen peroxide to a buffer [53, 54]. In both cases, oxidation of dopamine is performed by forming an intermediate copper (II)-o-quinolate complex (Fig. 11). Thus, these sensors respond to molecules with at least two hydroxyl groups in the ortho-position, such as catechol, dopamine, and pyrogallol, but not to phenol with only one hydroxyl group nor resorcinol and hydroquinone with two hydroxyl groups in meta- and para-positions, respectively. It clearly shows that the oxidation mechanism is different from that presented by Del Pilar et al. and Dos Santos et al. Aiming to boost a sensor’s selectivity, the influence of some o-diphenols can be eliminated by modifying an electrode with a polyanionic membrane such as Nafion®. As presented by Boulkroune et al., the polyanionic surface of their sensor’s does not exhibit affinity towards o-diphenols such as tiron, L-dopa, and 3,4-dihydroxybenzoic acid, possibly due to the repulsive effect on carboxylate or sulfonate groups. The interference of uric acid and ascorbic acid was investigated. Although at concentrations close to that of dopamine (130 µM interferant vs 105 µM dopamine) no significant change in response was observed, the signal dropped by 80% at the 12 × excess of each interferant.

Although most phenol sensors operate in neutral conditions, Khalid et al. have proven that catechol determination is also possible in 0.1 M NaOH (pH ~ 13), presenting a device based on CuO nanoparticles immobilised in a porous SiO_2_/graphite matrix [57]. As mentioned in the section dedicated to glucose detection, at alkali pH, copper is converted to copper oxyhydroxide; hence, there is no requirement for hydrogen peroxide as in the case of sensors designed by Dos Santos et al. and Del Pilar et al. The sensor response is not affected by the presence of 4-aminophenol, resorcinol, ascorbic acid, uric acid, sodium nitrate, or hydroquinone at the concentration of up to 3 M. An interference study did not include glucose, as it was not relevant for the sensor application in water analysis. However, glucose should be considered when analysing other fluids, as it results in a current increase at the same or very similar potentials.

## Iron and iron oxides

Most peroxidases contain an iron atom in a heme form at their active site; thus, this metal’s catalytic properties as an alternative to an enzyme have been broadly studied. In the active site of horseradish peroxidase, Fe^3+^ (heme) is oxidised to Fe^4+^ (oxo-ferryl) by hydrogen peroxide and then reduced back to its native form with the simultaneous oxidation of an electron donor species, such as phenol [58]. In electrochemical determination, an oxidised species is subsequently reduced at an electrode surface, resulting in a current proportional to an analyte concentration. Santos Damos et al. constructed a phenolic compounds sensor based on iron(III) tetra-(N-methyl-4-pyridyl) porphyrin (Fe III T4MpyP) in combination with histidine. This amino acid appears in close proximity to the heme centre of many peroxidases, where it serves as a stabiliser of higher oxidation states. At the optimum pH 4, the sensor responds to aromatic amines, with a higher signal observed for compounds with functional groups in positions para- and ortho-. On the other hand, there is no response to nitrophenols with electron-withdrawing substituents. Although the sensor was characterised in terms of catechol, it is suitable for dopamine determination, which gives an almost threefold signal. Due to the sensor’s cross-reactivity and inability to differentiate between various analytes, it is not suitable for detecting a single type of phenolic compound.

Hydrogen peroxide determination in PB at pH 7 was achieved using Fe_3_O_4_ magnetic nanoparticles [40–42]. Xiong et al. prepared a sensor based on Fe_3_O_4_ nanoparticles embedded in rGO. CV analysis showed that in the absence of H_2_O_2_, a pair of current peaks is attributed to a redox couple Fe^2+^/Fe^3+^, whereas the analyte’s addition leads to the cathodic peak increase and the anodic peak decrease [40]. The authors explain this by an EC mechanism (where E stands for an electrochemical and C a chemical reaction), in which an electrochemical reduction of Fe^3+^ to Fe^2+^ is followed by a chemical reduction of H_2_O_2_ by Fe^2+^.

Xia et al. presented a FeOOH nanowire-modified sensor for glucose determination at pH 7.4, in contrast to most of the glucose sensors described in the literature that operate in alkali conditions [36]. They showed that the oxidation peak current increases linearly upon increasing glucose concentration. The authors propose that ferrous and ferric ions play a key role in the process. The oxidation was proved to be proton-facilitated, as the anodic peak potential moves towards more negative values with the increase of pH. Dopamine and ascorbic acid were tested as possible interferants. Although both molecules lead to another peak formation during CV measurements, the potential difference between the peaks allows for complete differentiation of signals arising from the analyte and an interferant.

A superoxide dismutase-like activity of iron in the form of FePO_4_ was applied by Wang et al. to monitor O_2_^●–·^ released by living cells at pH 7.4 [66]. Amongst molecules such as ascorbic acid, H_2_O_2_, uric acid, and a few other carbohydrate compounds, only the first two affected the response, which was neglected for the study. Nonetheless, even though the interference is low, it should be considered depending on the sensor’s application.

Finally, an enzyme-like activity of a complex of iron with 1,3,5-benzenetricarboxylic acid (Fe(II)–BTC) was applied by Huang et al. for monitoring a NO photorelease from sodium nitroprusside [67]. NO is oxidised to NO^+^ with a simultaneous reduction of Fe(III) to Fe(II). Fe(II) is then re-oxidised and is ready to catalyse another NO molecule. CV performed at pH 7.4 in the absence of NO shows a pair of peaks attributed to the Fe(II)/Fe(III) redox couple, whereas in the presence of the analyte, another anodic peak appears. The extra peak indicates NO oxidation, which then undergoes further conversion, first to NO_2_^−^ and finally to NO_3_^−^. Hence, the oxidation reaction is irreversible, and no reduction peak is observed on a reverse scan. No significant change of the sensor’s response was recorded in the presence of an equal concentration of dopamine (6% signal change) nor ascorbic acid and uric acid (less than 5% signal change) at concentrations ten times greater than that of dopamine.

## Noble metals

Li et al. studied a peroxidase-like and a catalase-like activity of gold, palladium, platinum, and silver [72]. They concluded that at high pH, when hydroxyl groups are pre-absorbed onto a metal surface, hydrogen peroxide is decomposed in an acid-like manner, manifesting the catalase-like activity of a metal:

$${\mathrm{H}}_{2}{\mathrm{O}}_{2\left(\mathrm{ads}\right)}\leftrightarrow {\mathrm{H}}_{\left(\mathrm{ads}\right)}+{\mathrm{HO}}_{2\left(\mathrm{ads}\right)}$$

where the subscript *(ads)* indicates a species adsorbed on a metal.

On the other hand, at low pH, with H groups pre-adsorbed, a metal exhibits the peroxidase-like activity, decomposing H_2_O_2_ in a base-like based manner:

$${\mathrm{H}}_{2}{\mathrm{O}}_{2\left(\mathrm{ads}\right)}\leftrightarrow {\mathrm{H}}_{2}{\mathrm{O}}_{\left(\mathrm{ads}\right)}+{\mathrm{O}}_{\left(\mathrm{ads}\right)}$$

It was also shown that at neutral pH, the latter mechanism has a lower energy barrier; thus, it is favourable.

The nature of the oxidase-like activity of gold, palladium, platinum, and silver was explored by Shen et al. [9]. A triplet oxygen atom (^3^O_2_) contains two unpaired spin-parallel (spin-up) electrons in a π^*^ molecular orbital, making it unable to react directly with an organic substrate. When a triplet oxygen atom is adsorbed and dissociated on a metal surface, two spin-down electrons are transferred from a metal to a π^*^ molecular orbital of oxygen, resulting in excited, highly reactive singlet oxygen. Shen et al. proved that the oxidase-like activity of Au nanoparticles is facet-dependent. For example, Au(110) and Au(111) are characterised by low catalytic activity in contrast to high-energy facets such as Au(211), which are abundant in 3–5 nm nanoparticles or smaller. Despite a high-energy barrier of adsorption and dissociation of a triplet oxygen atom on Au(111) as well as Ag(111), the alloy of these two significantly reduces the energy barrier, resulting in the high oxidase-like activity of the alloy. The opposite happens for (111) facets of AuPd and AuPt alloy — a high catalytic activity of monometallic Pd and Pt is lowered upon combining with Au(111).

In terms of a superoxide dismutase-like activity of all four noble metals, a dismutation of superoxide anion is favoured and occurs through the protonation of O_2_^●–^ as follows:

$$O_2^{\bullet -}+H_2O\leftrightarrow{HO}_2^\bullet +{OH}^-$$

OH^–^ is readily adsorbed onto a metal’s surface and is subsequently rearranged, resulting in O_2_ and H_2_O_2_, which in turn is decomposed either in a base- or acid-like manner due to the peroxidase- and oxidase-like activity of metals, respectively.

### Gold

Maluta et al. proposed a sensor based on Au nanoparticle-decorated multi-walled carbon nanotubes (MWCNT) for NO determination at optimised pH 4.4 [68]. CV analysis revealed that the electrode can oxidise NO, which is manifested by an anodic peak at a potential + 0.98 V vs Ag/AgCl, not observed in the case of a bare GCE. It should be noted that non-decorated MWCNT also showed a response towards NO; however, the oxidation peak occurred at a higher potential (+ 1.18 V), and it was not as well-defined as in the case of Au NP/MWCNT/GCE. The electrode was further modified with a Nafion® membrane that inhibits further NO^+^ conversion to NO_2_^−^ and NO_3_^−^ by stabilising cationic species. The response was not affected by ascorbic acid, dopamine, uric acid, carbon dioxide, or oxygen gas. Although three extra peaks occurred in a voltammogram for the first three molecules, they did not overlap with the NO oxidation peak.

Gold nanoparticles immobilised on carbon nanostructures were also applied for hydrazine sensing. For this purpose, Zhao et al. functionalised a GCE with Au nanoparticle-decorated single-walled carbon nanohorns (SWCNH). Hydrazine electrooxidation at a bare electrode requires a large overpotential and results in a weak current peak. In contrast, a well-defined peak is observed with Au/SWCNH/GCE and at a significantly reduced potential (+ 0.15 V vs SCE)[61]. Although the SWCNH/GCE (without Au nanoparticles) did not show a substantial response increase compared to the bare electrode, the authors claim that SWCNH play a vital role in the oxidation process by creating a favourable microenvironment. Measurements were performed in an N_2_-saturated PBS at the optimised pH 7.4, which is, interestingly, different from the optimum pH for copper-based hydrazine oxidation found by Heydari et al. (pH 5). Possible interferants such as Na^+^, K^+^, NO_3_^–^, CO_3_^2–^, SO_4_^2–^, sodium citrate, urea, glucose, and oxalic acid were shown not to affect the sensor’s response.

It has been proven that Au nanoparticle-modified rGO exhibits a higher catalytic activity compared to Au nanoparticles or rGO separately due to their synergistic effect. First, it can be explained by a strong interaction between orbitals 5d and 2p of a gold and carbon atom, respectively, desirable for the absorption of H_2_O_2_ and HO^●^. Secondly, n-doping of rGO attributes to its electronic structure and Ferni level, enhancing the catalysis [73]. This phenomenon was utilised by Bilgi Kamac et al., who combined Au NP/rGO with a poly(neutral red) conductive polymer that served as a redox mediator in a hydrogen peroxide sensor [43]. At the optimised pH 7, the response was not affected by NADH, uric acid, dopamine, and ascorbic acid (100 µM each).

### Palladium

The catalytic activity of palladium has been widely studied for sensing applications, and it is shape- and size-dependent. A metal oxide layer on the surface of Pd nanoparticles is reduced in the presence of H_2_O_2_, followed by electrochemical re-oxidation.

Patella et al. compared responses of Pd thin film, Pd nanoparticles (NP), and Pd nanowires (NW) of different lengths (5.1 µm, 2.1 µm, and 1.7 µm) to H_2_O_2_ at pH 7 [44]. Due to nanowires’ hydrophobic nature, all experiments were performed in alcohol solutions to increase nanostructures’ wettability. It was clear that a higher surface area of nanostructured Pd compared to Pd thin film (and Pd NW compared to Pd NP) leads to improved sensing characteristics. Although the length of nanowires seemed not to affect the lower end of a linear range, the upper limit increased with nanostructures’ length, meaning that shorter NW become saturated at lower analyte concentrations. However, a LOD decreased slightly, from 17.1 µM for 1.7 µm NWs to 13.3 µM for 5.1 µm NWs. 5.1 µm NW were chosen as the best performing hydrogen peroxide reduction catalyst due to the broad linear range and the highest sensitivity. Although the optimisation study showed that the most significant change in current upon addition of H_2_O_2_ is achieved at − 0.25 V vs Ag/AgCl, a lower potential of − 0.2 V was used to ensure the sensor’s selectivity. During chronoamperometric analysis, no response was observed for 1 mM of glucose, ascorbic acid, potassium oxalate, or uric acid. On the other hand, upon adding 10 mM of each molecule, a sudden variation of the signal occurred, followed by its return to the initial value (from before the addition of an interferant).

Karakaya et al. designed a flow-injection H_2_O_2_ sensor based on a Pd nanoparticle-modified pencil graphite electrode [45]. CV measurements at pH 7 showed that the electrode’s functionalisation with Pd nanoparticles reduced a cathodic peak potential by 4.5 times and increased its current by approximately 10–15 times. Amperometric optimisation tests revealed that the reduction current increases with a decreasing potential in the range from 0 to − 0.6 V vs Ag/AgCl. Despite that, − 0.1 V was chosen as a working potential, at which the current was still much higher compared to a nonmodified electrode (by 187-fold) and which minimises the interference of dissolved oxygen when not using an argon-saturated supporting electrolyte. The sensor sensitivity is 12.606 mA mM^−1^ compared to 0.037 mA mM^−1^ of a nonmodified electrode. An additional coating of the sensor with a Nafion® membrane successfully quenched the interference of ascorbic acid, dopamine (at 1:1 ratio of an interferant to the analyte) and uric acid (at the ratio of 2:1 of an interferant to the analyte).

**Fig. 12:**
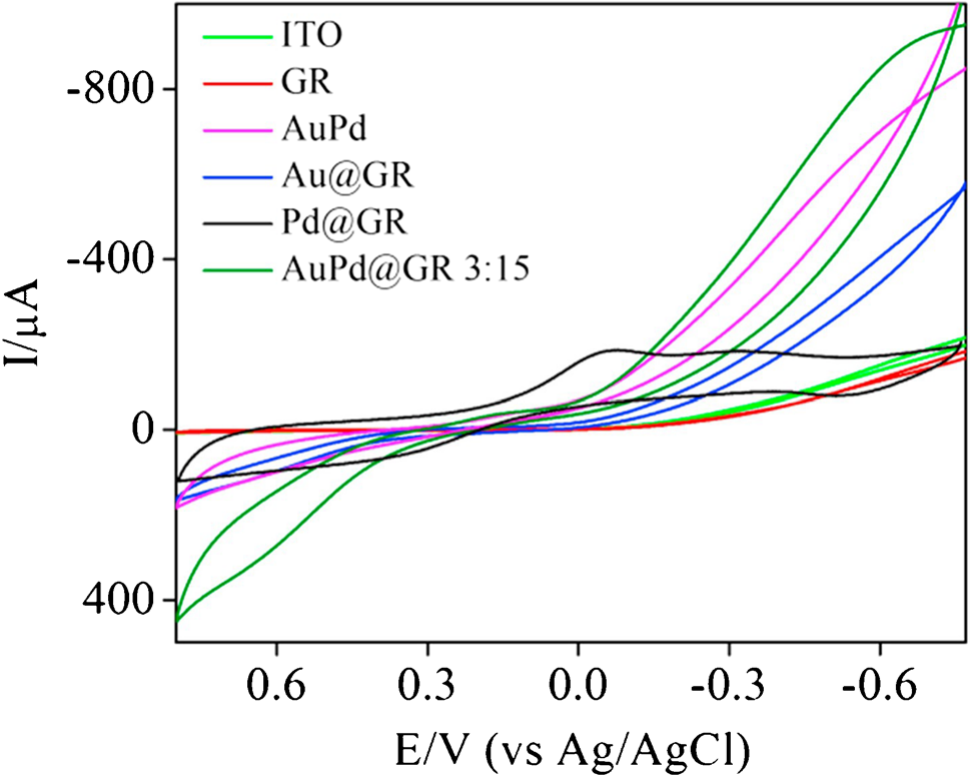
CVs recorded in a 0.1 M PBS containing 5 mM H_2_O_2_ using different modified ITO electrodes. Figure reproduced with permission from ref. [46]; copyright © 2016 Elsevier B.V

Thanh et al. encapsulated ~ 5 nm AuPd nanoparticles in graphene. The authors ascribe the desirable sensing performance to Au^3+^ and Pd^2+^ predominantly present as active catalytic sites [46]. CV analysis performed in an N_2_-saturated supporting electrolyte containing H_2_O_2_ at pH 7.4 shows two peaks, the anodic one at around + 0.6 V and the cathodic one at around − 0.6 V vs Ag/AgCl. AuPd nanoparticle-modified graphene electrode exhibits a significantly higher reduction current than when modified only with either Au or Pd nanoparticles, proving the synergistic effect of these metals (Fig. 12). The selectivity of the sensor was confirmed by the lack of response to 0.1 mM uric acid, 0.5 mM glucose, 0.05 mM K^+^, 0.05 mM Cl^–^, 0.1 mM ascorbic acid, 0.1 mM urea, and 0.005 mM dopamine.

**Fig. 13:**
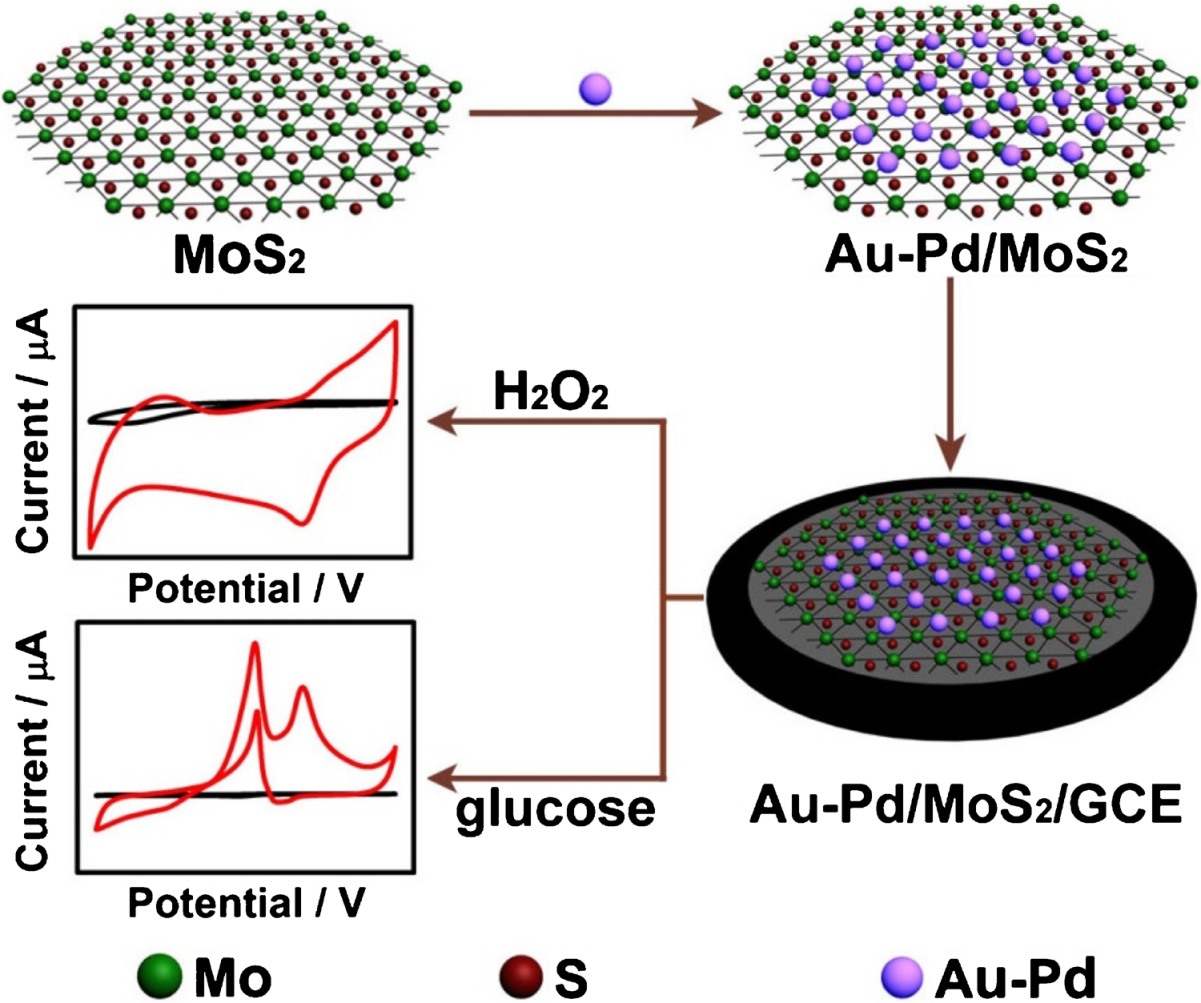
Schematic illustration of Au–Pd/MoS_2_/GCE fabrication for the determination of H_2_O_2_ and glucose. Figure reproduced with permission from ref. [37]; copyright © 2016 Elsevier B.V

Similarly, Li et al. designed a sensor based on AuPd nanoparticles anchored in molybdenum disulphate nanosheets (AuPd/MoS_2_/GCE) (Fig. 13) [37]. By changing the pH of a supporting electrolyte from 13 to 7.4, the authors modulated a nanocomposite selectivity from glucose to hydrogen detection, respectively. A CV analysis performed at neutral pH revealed that neither Au nor MoS_2_ on their own has the ability to catalyse H_2_O_2_ reduction. Exclusively Pd-containing electrodes show a cathodic response to the analyte, but only AuPd/MoS_2_/GCE provides a strong electrocatalytic signal. The authors explained that although Au and MoS_2_ do not have catalytic activity towards H_2_O_2_, they boost the sensor’s response by promoting a fine dispersion of Pd. With 2 mM KCl, NaNO_3_, Na_2_SO_4_, and 4-acetamidophenol, no interference was detected with the DPV-based H_2_O_2_ determination. Negligible interference of 0.4 mM ascorbic acid and uric acid at 0.4 mM could be eliminated by incorporating a Nafion® membrane. Regarding glucose determination, CV analysis at pH 13 showed that both Au and Pd exhibit a catalytic activity towards glucose oxidation with a much stronger response of the latter. As in the previous application of the sensor, the synergistic effects of the three nanostructures (Au, Pd, and MoS_2_) was also confirmed for glucose determination. At the potential − 0.1 V vs SCE, the glucose sensor does not respond to 5 mM KCl, Na_2_CO_3_, Na_2_SO_4_, and lysine, and only an insignificant response is observed in the presence of ascorbic acid and uric acid. Conversely, 0.05 mM mannose, fructose, galactose, sucrose, and lactose interfere with glucose detection.

As mentioned in the previous section, Shen et al. concluded that the high catalytic activity of monometallic Pd is quenched when mixed with Au (111) [9]. Nevertheless, both studies of Li and Du and of Thanh et al. prove the synergistic effect of Au and Pd in bimetallic alloys, which indicates the importance of molybdenum disulphate nanosheets and graphene in the overall activity of a composite. Both supporting materials have been shown to be able to reduce the size of synthesised metal nanoparticles. Dominant crystal planes of bulk metals as well as of large nanoparticles are (111) and (110), which are the most stable, hence the least reactive due to a high coordination number of atoms exposed on a surface. Upon shrinking nanoparticles, more atoms at less stable facets are exposed, and similarly, atoms are exposed at even less coordinated sites such as boundaries between surface planes, which enhances the reactivity of nanoparticles [74]. In other words, the discontinuity of crystal planes is an essential factor affecting nanoparticles reactivity.

Finally, Pd nanoparticles were applied for hydrazine determination in a N_2_-saturated electrolyte at pH 7 [62]. Cobalt and nitrogen-doped carbon nanotubes were used as support for Pd nanoparticles, ensuring their uniform dispersion. Palladium catalytic activity was confirmed by an increasing current of a hydrazine oxidation peak with an increasing Pd content in a sensing nanocomposite. Details of the sensor’s analytical performance can be found in Table 4. Amongst the molecules studied were uric acid (4:1 ratio), Na^+^, CO_2_^−^ (40:1 ratio), glucose, fructose, NH_3_·H_2_O, NH_4_^+^, Cl^−^, K^+^, NO_3_^−^, and Br^−^ (100:1 ratio).

### Platinum

Zhang et al. utilised platinum nanoparticles to design a bifunctional sensor for glucose and hydrogen peroxide determination [38]. High dispersion of Pt nanoparticles was achieved by confining them into a mesoporous structure of hollow carbon spheres. In an N_2_-saturated buffer at pH 7.4, platinum provides active sites for H_2_O_2_ reduction.

As seen in the previous examples, glucose detection is usually performed in an alkali environment; however, Zhang et al. executed it at pH 7.4. A mechanism of glucose oxidation was studied using CV. Firstly, glucose is adsorbed onto a surface of Pt nanoparticles, which is electrochemically manifested by an anodic peak at a potential − 0.4 V vs Ag/AgCl. Next, it is oxidised to gluconic acid, giving rise to another anodic peak at + 0.04 V. However, the product accumulating on the sensing surface limits the oxidation process. Eventually, when the potential of Pt oxidation is reached (+ 0.4 V), generated Pt-OH can further oxidise glucose to gluconic acid and gluconic acid to gluconolactone, to which an anodic peak at + 0.6 V is attributed. Therefore, for amperometric glucose determination, + 0.6 V was chosen as a working potential. No significant response was observed to sucrose, lactose, or maltose (at 2 mM each), proving the sensor’s selectivity.

### Silver

Hydrogen peroxide detection is by far the most exploited application of silver nanoparticles in sensors. Koyappayil et al. studied both a peroxidase-like and a catalase-like activity of Ag nanoparticles, showing that the optimum pH for these activities is pH 3 and 6, respectively [47], which is in contradiction to the study presented by Li et al. [72]. An amperometric H_2_O_2_ determination was performed in acidic conditions at a working potential − 0.165 V vs SCE. However, low pH is not always compatible with analysed samples, for example, serum.

He et al. loaded Ag nanoparticles onto Na_2_Ti_3_O_7_ nanowires using graphite as a substrate and performed an H_2_O_2_ determination at pH 7.4 [48].

The mechanism of hydrogen peroxide oxidation mediated by Ag nanoparticles is suggested to be similar to the Pd nanoparticle-mediated reaction. Existing on the surface of Ag nanoparticles, argentous oxide is first reduced by hydrogen peroxide and then re-oxidised at an electrode:

$${2\mathrm{Ag}}_{\mathrm{ox}}+{\mathrm{H}}_{2}{\mathrm{O}}_{2}\to 2\mathrm{Ag}+{2\mathrm{H}}^{+}+{\mathrm{O}}_{2}$$

$$\mathrm{Ag}\to {\mathrm{Ag}}_{\mathrm{ox}}+{\mathrm{e}}^{-}$$

On the other hand, the mechanism of a hydrogen peroxide reduction is explained by the Haber–Weiss O_2_^2−^-mediated reaction, in which hydroxyl radicals are generated:

$${\mathrm O}_2^{2-}+{\mathrm H}_2{\mathrm O}_2\rightarrow{2\mathrm{OH}}^-+\mathrm{OH}^\bullet +{\mathrm O}_2$$

$$\mathrm{OH}^\bullet +\mathrm e^-\rightarrow\mathrm{OH}^-$$

Zhao et al., who loaded Ag nanoparticles onto MWCNT, used a cathodic current analysis for H_2_O_2_ determination [49]. Despite the highest response at − 0.45 V vs Ag/AgCl, − 0.2 V was chosen as a working potential, which eliminated possible interference from co-existing biomolecules, such as ascorbic acid, uric acid, and acetaminophen (at a concentration of 0.1 mM), as well as limited an oxygen reduction current.

## Challenges and outlook

Metal/metal oxide nanoparticles can serve as a cheaper and more robust alternative to enzymes. Although numerous examples of nanozyme-based biosensors exist in the literature, most commercialised devices still rely on enzymes. The three-dimensional structure of their active sites results in excellent specificity and sensitivity. These two parameters need to be improved in the case of metal/metal oxide nanoparticles to rationalise their use as a replacement for enzymes. Poor selectivity of metal/metal oxide nanoparticles is prominent. In alkali conditions, copper, nickel, and their alloys have been shown to catalyse the conversion of different analytes, including glucose, hydrazine or hydrogen peroxide. Similarly, copper can be used to detect glucose and catechol, both at pH 13. In a neutral environment, its affinity is shifted towards hydrogen peroxide, various phenolic compounds, hydrazine, and superoxide anion. These examples highlight the issue of unspecific interactions of nanozymes with multiple analytes in the same conditions, which has to be addressed. An in-depth understanding of the relationship between the sensor’s catalytic activity and an analyte, sensing conditions, and nanoparticles composition and structure is required to achieve that goal.

Many of the research papers that describe the development of a metal/metal oxide nanoparticle-based sensor do not provide an insight into the principles of analyte detection. Discrepancies amongst the papers that explain the detection mechanism lead in some cases to confusion and inconclusive findings. Conversely, the reviews on nonenzymatic devices tend to focus on their analytical performance without relating it to the existing knowledge of metals’ catalytic activity and the factors affecting it. On the other hand, the reviews on the principles behind the enzyme-like activity of metal/metal oxide nanoparticles often lack a sufficient number of examples from the application-focused research papers. Bridging this gap between fundamental and applied research is necessary to obtain a complete picture of nanoparticles perspectives and challenges in biosensors.

Improving the catalytic properties of metal/metal oxide nanoparticles is mainly achieved by trial and error. However, a more evidence-based approach and the implementation of theoretical studies can facilitate the process. Multiple factors contribute to superior enzyme characteristics, understanding which will help to mimic enzymatic properties using nanozymes. For example, the possibility of conjugating metal/metal oxide nanoparticles with amino acids commonly found in enzymes’ active sites should be explored. Conjugating nanoparticles with synthetic molecular recognition elements with an affinity towards an analyte of interest, such as aptamers or molecularly imprinted polymers (MIP), is also promising in enhancing the selectivity of nanozymes.

A common way of circumventing the interference of negatively charged species is using a Nafion coating, which repels anionic species from an electrode surface. However, it significantly reduces the electrical conductivity. Alternative membranes with improved properties and broader application, for example, to repel positively charged interferants, are in demand.

Although metal/metal oxide nanoparticles meet many of the POC requirements challenging to satisfy by enzymes, such as stability and cost-effectiveness, more multidisciplinary research is required for nanozymes to successfully replace their protein equivalents in real-life applications.

## Concluding remarks

This review underlines the complexity involved in the process of biosensor design, for which there is no universal guideline. To detect an analyte of interest, one must choose the most appropriate metal with a desired enzyme-like catalytic activity, noting that metal oxide or metal alloys may be a better option in some circumstances. For example, Rahim et al. designed a glucose biosensor and proved that copper oxide nanoparticles performed better than metallic copper nanoparticles [30]. Shen et al. performed experiments showing that although neither Au (111) nor Ag (111) serves as a good oxidase mimetic, their alloy exhibits a high oxidase-like activity [9].

The synthesis process is an essential step as it determines the size and shape of metal nanoparticles, which govern their catalytic properties. Besides the active surface area and the accessibility of catalytic sites, reactivity is dependent on crystal planes by which nanoparticles are bound. Shrinking a nanoparticle favours the existence of high-index crystal planes, enhancing its overall catalytic reactivity. However, it has been shown that the difference in activity can be significant for nanoparticles of varying shapes despite the same size. For example, platinum tetrahexahedral nanoparticles exhibit 400% catalytic activity of similar size platinum spherical nanoparticles [75]. Thus, a shape- and size-controlled synthesis of nanoparticles is the key to get the most of nanoparticles’ catalytic properties. Both parameters can be tuned by varying synthesis temperature, precursors, and reducing as well as stabilising agents, and solvents. Another critical factor to consider is the choice of supporting material on/in which nanoparticles are embedded. Carbon-based substrates such as graphene dots reduced graphene oxide, single- or multi-walled carbon nanotubes but also molybdenum disulphate nanosheets have been shown to promote fine nanoparticles dispersion.

Experimental conditions such as temperature, pH, solvents, and a working potential need to be optimised for the specific application of interest. In terms of temperature, it is commonly known that higher temperature increases particles’ motion speed and, therefore, intermolecular interactions. However, in some applications, the elevated temperature is not feasible, for example, due to the thermal instability of an analyte or another sample/device component. Managing temperature requires an extra control system, increasing an overall design complexity, which should be avoided for point-of-care applications. The pH of an environment is likely to be one of the most important parameters, and it depends on a mechanism of a reaction catalysed by metal nanoparticles. The pH of a solution determines a surface charge; it affects the reactivity of the analyte; it changes the behaviour of interferences and therefore strongly alters interactions between surface and molecular species. A good example is an effect of pH on a hydrogen peroxide decomposition, which can be performed either in a catalase- or a peroxidase-like manner in an acidic or an alkali environment, respectively. From Tables 2, 3, and 4, it can be noted that glucose detection is nearly always performed at alkali pH, whereas dopamine and other phenolic compounds are detected at pH ranging from slightly acidic to neutral, regardless of the material used as an enzyme mimetic. The solvent choice seems somewhat less critical, provided it meets standard requirements for an electrolyte, such as inertness, high conductivity, and an appropriate buffering capacity. A deaerated buffer is often used to avoid oxygen interference and is obtained by sparging it with nitrogen, argon, or other inert gasses. An amperometric sensor’s working potential is another important parameter to be optimised. While looking for the highest response, it must be kept in mind that at a specific potential, more than one molecule can undergo oxidation or a reduction influencing the outcome signal.

We here provided a comprehensive review of numerous biosensors based on metal and metal oxide nanoparticles as an enzyme mimetic component. It depicts the nanomaterial behaviour in different designs and environmental conditions, highlighting the importance of the above on its catalytic activity. The application of nonenzymatic sensors is highly desirable for point-of-care testing as it overcomes the issues of enzyme stability, which can hinder the sensor application in different settings (e.g. low-resource settings without suitable temperature and humidity control) and increase their shelf-life. Different nanomaterials will be suitable for the detection of different analytes but of key importance is the careful design of the sensors and of the measurement conditions in order to achieve selective catalytic properties. By tuning the experimental conditions of the measurements, improved selectivity can be achieved with nanomaterial-based catalytic sensors, making them applicable to point-of-care or lab-on-chip devices.
